# 

**Published:** 1904-09-17

**Authors:** 


### NOTICE TO CORRESPONDENTS.

All MSS., Letters, Books for Review, and other matters intended for the Editor should be addressed The Editor, "The Hospital," The Hospital Buildings, 28 & 29 Southampton Street, Strand, W.C.

The Editor cannot undertake to return rejected MS., even when accompanied by stamped directed envelope.

**Notice to Advertisers and Subscribers.**

All Advertisements, Orders for Copies of The Hospital, and Business Communications should be addressed to **THE MANAGER (Not to the Editor),** The Hospital, 28 & 29 Southampton Street, Strand, London, W.C.

The Cost of Subscription, post free, is as follows:—Per week, 3½d.; per quarter, 3s. 3d.; per half-year, 6s. 6d.; per annum, 13s. Foreign and Colonial:—Per annum, 19s. 6d., payable, in all cases, in advance.

**NOTICE.—Vol. XXXV. of "The Hospital" (October 1903 to March 1904), in handsome cloth binding, is now on sale at the office of this Journal, price 10s. 6d. Cases for binding the Numbers contained in this Volume 2s. Index to Vol. XXXV., 3½d., post free.**

**The Monthly part for September is now ready. Price 1s., by post, 1s. 3d.**

### BOOKS RECEIVED.

The Vegetarian Society.

"A Dozen Plain Dinners Plainly Cooked."

The Institution of Heating and Ventilating Engineers.

"Mechanical Draught for Boilers." By Walter Yates.

Metropolitan Asylums Board.

"Annual Report, 1903."

McDougall's Educational Company, Limited.

"Laws of Health."

Macmillan and Co.

"The Story of an East London Hospital."

Spiers & Pond, Limited.

"Lemco Dishes for all Seasons."

Adlard & Son.

"The Differential Diagnosis of Syphilitic and Non-syphilitic Affections of the Skin." By G. Pernet.

Rebman, Limited.

"Health and Disease in Relation to Marriage and the Married State." By Senator and Kaminer.

R. Burnet, M.B., Ch.B.Vict., District Medical Officer of the Parish of Birmingham. W. H. Carse, M.B., C.M.Edin., District Medical Officer of the Rochdale Union. J. A. Codd, M.D.Lond., B.Sc., Medical Officer of Health, Heath Town Urban District. H. A. Cruttwell, L.R.C.P.&S.Edin., L.F.P.S.Glasg., District Medical Officer of the Chertsey Union. Arthur M. Cudmore, M.B., F.R.C.S., Surgeon to the Adelaide Hospital, South Australia. T. H. L. Cumpston, M.B., B.S., Resident Medical Officer, Parkside Lunatic Asylum, South Australia. R. S. C. Edleston, M.R.C.S., L R.C.P.Lond., District Medical Officer of the Bakewell Union. W. C. Edmonston, M.B., C.M.Aberd., District Medical Officer of the Pembroke Union. J. G. Flannery, L.R.C.P. & S.I Certifying Factory Surgeon for the Tubercurry District, Count Sligo. S. G. Graham, M.R.C.S., L.R.C.P.Lond., Medical Offic of Health for the Watchet Urban District. O. W. Griffitł L.R.C P. & S.Edin., District Medical Officer of the Pwllheli Union T. A. Hawkesworth, M.D.Lond., M.R.C.S.Eng., District Medical Officer of the Henley Union. William Shaw, M.D Honorary Physician to the West Kent General Hospital, Maic stone. William H. Wynn, M.D., B.Sc.Lond., M.Sc., M.B B.Ch.Birm., Casualty Assistant Physician to the General Hospita Birmingham.

**"The Hospital" Institutional Library.**

"Hospitals and Asylums of the World." 4 Vols., with a Port fclio of Plans, complete £12 12s.

"Burdett's Hospitals and Charities, 1904." 5s. net.

"Burdett's Official Nursing Directory." 8s. net.

"Cottage Hospitals, General, Fever, and Convalescent." 10s. 6d

"Uniform System of Accounts for Hospitals and Public Institu tions." New Edition. 4s. net.

"Hospital Expenditure: The Commissariat." 2s. 6d.

"A Legal Handbook for the Use of Hospital Authoriti 2s. 6d.

"The Cottage Hospital Case Book or Register of Patients." 1 and 12s. 6d.

All these are published by the Scientific Press, Ltd., and m be obtained through any bookseller, or direct from the publisher 28 and 29 Southampton Street, Strand, London, W.C.

THE BEST NATURAL APERIENT WATER.

### Hunyadi János

**For LIVER COMPLAINTS, OBESITY, &c.**

The "VIENNA MEDICAL PRESS" says:—

**"Hunyadi János may be regarded as a specific for obesity."**

Average Dose.—*A wineglassful before breakfast, either pure or diluted with a similar quantity of hot or cold water.*

**CAUTION.**—Note the name "Hunyadi János," the signature of the Proprietor, ANDREAS SAXLEHNE and the Medallion, on the Red Centre Part of the Label.

### A Dozen Books every Nurse should Possess.

| | | |
|---|---|---|
| 1. | **A HANDBOOK FOR NURSES.**<br>By J. K. WATSON, M.D.<br>Profusely Illustrated. Cloth gilt. | 5/- net,<br>5/4 post free. |
| 2. | **A COMPLETE HANDBOOK OF MIDWIFERY.**<br>By J. K. WATSON, M.D.<br>Illustrated with 143 Diagrams. Cloth gilt. | 6/- net,<br>6/4 post free. |
| 3. | **PRACTICAL GUIDE TO SURGICAL BANDAGING AND DRESSINGS.**<br>By WM. JOHNSON SMITH, F.R.C.S.<br>Suitable size for the apron pocket. Profusely Illustrated. Cloth. | 2/-<br>post free. |
| 4. | **SURGICAL WARD-WORK AND NURSING.**<br>By ALEXANDER MILES, M.D.Edin.<br>Second Edition. Illustrated. Cloth gilt. | 3/6 net,<br>3/10 post free. |
| 5. | **ART OF MASSAGE.**<br>By MRS. CREIGHTON HALE.<br>Second Edition Illustrated. | 6/-<br>post free. |
| 6. | **ELEMENTARY ANATOMY AND SURGERY FOR NURSES.**<br>By WM. McADAM ECCLES, M.S., M.B.<br>Profusely Illustrated. Cloth. | 2/6<br>post free. |
| 7. | **ELEMENTARY PHYSIOLOGY FOR NURSES.**<br>By C. F. MARSHALL, M.D, B.Sc., F.R.C.S.<br>Profusely Illustrated. Cloth. | 2/-<br>post free. |
| 8. | **NURSING IN DISEASES OF THE THROAT, NOSE, & EAR.**<br>By P. MACLEOD YEARSLEY, F.R.C.S., M.R.C.S.<br>Illustrated. Cloth. | 2,6<br>post free. |
| 9. | **OPHTHALMIC NURSING.**<br>By SYDNEY STEPHENSON, M.B., F.R.C.S.E.<br>New Revised Edition. Illustrated. | 3/6 net,<br>3/10 post free. |
| 10. | **THE EXAMINATION OF THE URINE.**<br>By J. K. WATSON, M.D.<br>Cloth boards. Suitable size for the apron pocket. | 1/- net,<br>1/1 post free. |
| 11. | **THE NURSES' PRONOUNCING DICTIONARY OF MEDICAL TERMS AND NURSING TREATMENT.**<br>Compiled by HONNOR MORTEN.<br>Fifth Edition, with Phonetic Pronunciation, Revised and Edited by MARY I. BURDETT. Suitable size for the apron pocket. Cloth. | 2/-<br>post free. |
| 12. | **FEVERS AND INFECTIOUS DISEASES.**<br>By WILLIAM HARDING, M.D.Ed.<br>Bound in cloth. | 1/-<br>post free. |

SEND FOR LATEST CATALOGUE OF NURSING MANUALS

*The above Books are obtainable of all Booksellers, or of*

**THE SCIENTIFIC PRESS, Ltd.,** 28 and 29 Southampton Street, Strand, London, W.C.

